# Dysregulated expression of *homeobox* family genes may influence survival outcomes of patients with epithelial ovarian cancer: analysis of data from The Cancer Genome Atlas

**DOI:** 10.18632/oncotarget.19771

**Published:** 2017-08-01

**Authors:** Kyung Jin Eoh, Hee Jung Kim, Jung-Yun Lee, Eun Ji Nam, Sunghoon Kim, Sang Wun Kim, Young Tae Kim

**Affiliations:** ^1^ Institute of Women’s Medical Life Science, Department of Obstetrics and Gynecology, Yonsei University College of Medicine, Seoul, Korea

**Keywords:** carcinogenesis, epithelial ovarian cancer, homeobox genes, survival, TCGA

## Abstract

*Homeobox (HOX)* family genes encode key transcription factors for embryogenesis and may be correlated with carcinogenesis. The aim of this study was to elucidate whether aberrant expression of *HOX* genes influences outcomes in epithelial ovarian cancer (EOC). Gene expression data and clinicopathologic information from 630 patients with EOC were downloaded from The Cancer Genome Atlas database. We explored correlations between expression levels of *HOX* gene family members and clinicopathological variables. Higher expression of *HOXA1*, *A4*, *A5*, *A7*, *A10*, *A11*, *B13*, *C13*, *D1*, and *D3* was associated with advanced FIGO stage. Suboptimal residual disease after debulking surgery was significantly correlated with higher expression of *HOXB9*, *B13*, and *C13*. Additionally, patients with high expression of *HOXC6* and *C11* were significantly more likely to have poor performance status. Overall survival was significantly shorter in patients with high, rather than low, expression of two *HOX* genes (*HOXA10* and *B3*), and significantly longer in patients with high rather than low *HOXC5* expression. Dysregulated expression of the *HOXA10*, *B3*, and *C5* was significantly correlated with overall survival in EOC patients. *HOX* gene expression levels are potentially useful as a prognostic indicator in EOC, and *HOX* genes may represent a novel and promising target for anticancer therapeutics.

## INTRODUCTION

Epithelial ovarian cancer (EOC), the leading cause of gynecologic cancer-related mortality, is known to be a multifactorial disease involving genetic, environmental, and epigenetic factors [1, 2]. Recently, research has focused on aberrant variation in the expression of transcription factors and its importance in the development of this malignancy [3].

*Homeobox (HOX)* genes, defined by a highly conserved 183-base pair DNA sequence called the homeobox, encode homeoproteins that function as transcription factors in differentiation and proliferation processes during embryonic structural development [4]. Aberrant expression of these proteins has been associated with carcinogenesis and aggressiveness [5, 6]. This is consistent with the idea that expression levels of many genes involved in normal embryogenesis are aberrant in various malignancies [7].

Previous studies showed that dysregulation of several *HOX* genes (including aberrant expression of *HOXA7*, *HOXA9*, *HOXC8, HOXB13*, and *HOXC6*) was associated with adverse prognostic factors in EOC; however, the majority of this research was limited by small sample sizes and was performed only *in vitro* [8–14]. Several studies have shown potential roles for *HOX antisense* long non-coding RNAs in ovarian cancer aggressiveness [15, 16]. These results suggest that aberrant expression of *HOX* genes may be correlated with the process of ovarian cancer carcinogenesis.

We analyzed the mRNA expression level of all 39 *HOX* genes using data from The Cancer Genome Atlas (TCGA) database on epithelial ovarian cancer and explored correlations with clinical data, including survival outcomes. We also evaluated the prognostic value of *HOX* gene expression analysis.

## RESULTS

In total, we included 630 ovarian cancer cases, which were all the patients who included in the TCGA ovarian cancer database. The median follow-up period was 34.0 months (range, 0.3–182.7 months). Among clinicopathological features, age at the time of diagnosis, advanced Federation of Gynecology and Obstetrics (FIGO) stage, suboptimal residual disease, and poor Eastern Cooperative Oncology Group (ECOG) performance status were found to be independent predictive factors for overall survival in multivariate Cox regression analysis (Table 1).

**Table 1:** Correlation between clinicopathological features and overall mortality

Variables	Total n = 630 n (%)	Multivariate analysis	
		HR (95% CI)	P
Age			
<45	54 (8.6)	1 (Reference)	0.042
≥45	553 (87.8)	1.512 (0.991-2.309)	
N/A	23 (3.7)		
Stage			
I	18 (2.9)	1 (Reference)	0.002
II	33 (5.2)		
III	462 (73.3)	2.264 (1.272-4.029)	
IV	89 (14.1)		
N/A	28 (4.4)		
Histology			
Serous	607 (96.3)		
N/A	23 (3.7)		
Grade			
1, 2	85 (13.5)	1 (Reference)	0.253
3, 4	505 (80.2)	1.189 (0.884-1.598)	
N/A	40 (6.3)		
LN metastasis			
No	85 (13.5)	1 (Reference)	0.125
Yes	139 (22.1)	1.387 (0.913-2.107)	
N/A	406 (64.4)		
Residual disease			
≤ 1 cm	387 (61.4)	1 (Reference)	<0.001
≤ 2 cm	151 (24.0)	1.548 (1.222-1.960)	
N/A	92 (14.6)		
ECOG			
0, 1	123 (19.5)	1 (Reference)	0.002
2, 3	12 (1.9)	3.317 (1.552-7.087)	
N/A	495 (78.6)		

N/A, not available; ECOG, The Eastern Cooperative Oncology Group performance status; HR, hazard ratio; CI, confidence interval; LN, lymph node.

Table 2 shows correlations between clinicopathological features and mRNA expression levels of *HOX* family genes. When the cases were categorized into low and high mRNA expression groups based on cut-off values determined as the median for each gene, higher expression of *HOXA1* (P = 0.011), *A4* (P = 0.045), *A5* (P = 0.01), *A7* (P = 0.047), *A10* (P = 0.009), *A11* (P = 0.001), *B13* (P = 0.01), *C13* (P = 0.022), *D1* (P = 0.004), and *D3* (P = 0.011) was associated with advanced FIGO stage. Suboptimal residual disease after debulking surgery was significantly correlated with higher expression of *HOXB9* (P = 0.002), *B13* (P = 0.008), and *C13* (P = 0.04). In addition, patients with high expression of *HOXC6* (P = 0.018) and *C11* (P = 0.049) were significantly more likely to have poor performance status (ECOG 2 or 3).

**Table 2:** Summary of the correlation between clinicopathological features and mRNA expression counts of homeobox (*HOX)* family genes

Age≥45		Stage III/IV	Grade 3/4	LN metastasis	Residual disease > 1 cm	ECOG 2, 3
*HOXA1*		+				
*HOXA2*						
*HOXA3*				+		
*HOXA4*		+				
*HOXA5*		+		+		
*HOXA6*						
*HOXA7*		+				
*HOXA9*						
*HOXA10*		++				
*HOXA11*		++		+		
*HOXA13*						
*HOXB1*						
*HOXB2*				++		
*HOXB3*			+			
*HOXB4*						
*HOXB5*						
*HOXB6*						
*HOXB7*						
*HOXB8*						
*HOXB9*					++	
*HOXB13*		+			++	
*HOXC4*						
*HOXC5*						
*HOXC6*						+
*HOXC8*						
*HOXC9*						
*HOXC10*						
*HOXC11*						+
*HOXC12*						
*HOXC13*		+			+	
*HOXD1*		++				
*HOXD3*		+	++			
*HOXD4*						
*HOXD8*						
*HOXD9*						
*HOXD10*						
*HOXD11*				++		
*HOXD12*				++		
*HOXD13*			+			

+, Correlation with P value < 0.05; ++, correlation with P value <0.01; LN, lymph node; ECOG, The Eastern Cooperative Oncology Group performance status.

**Figure 1:**
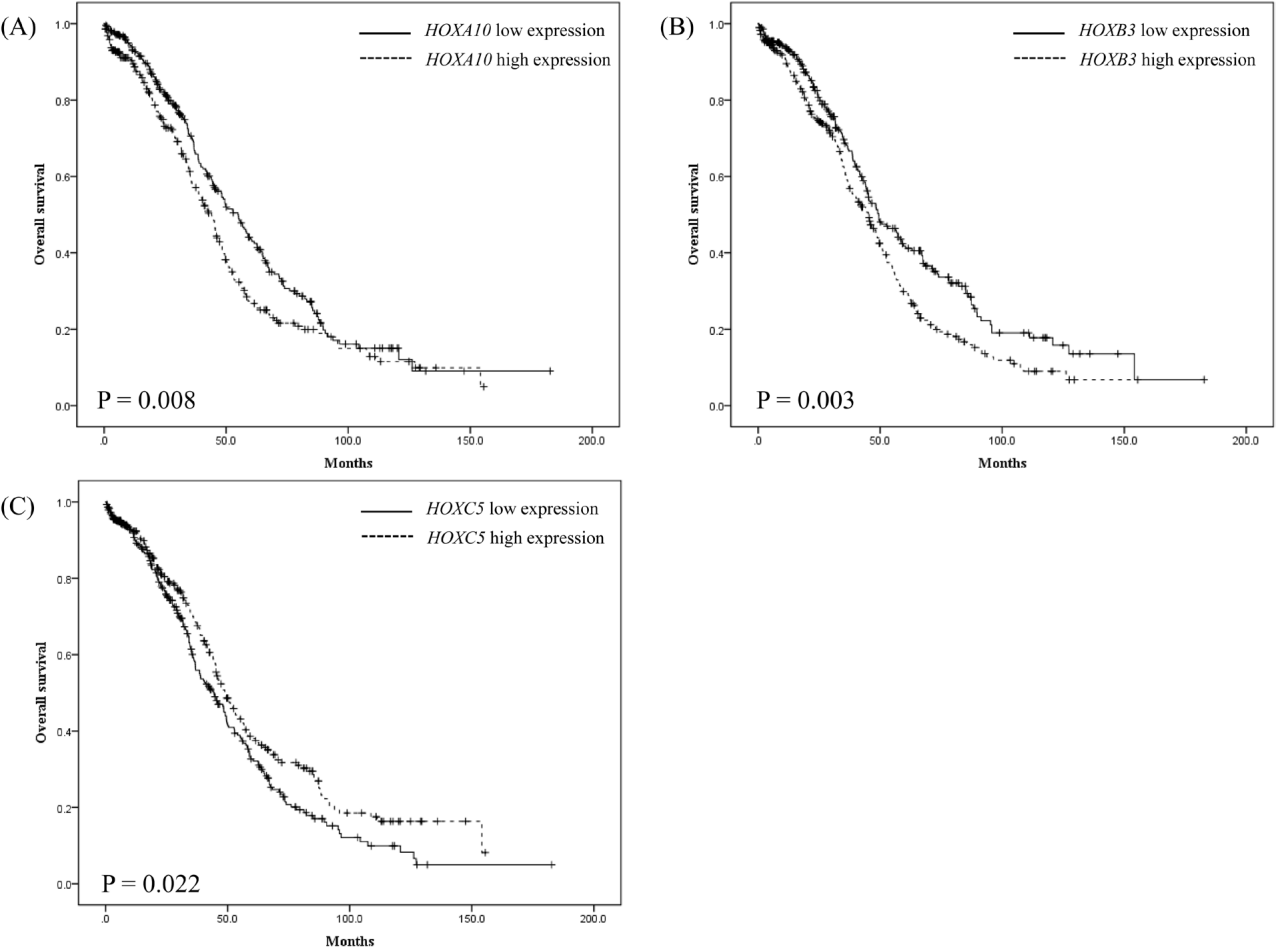
Kaplan-Meier survival analysis of 584 patients with ovarian cancer stratified by **(A)** *HOXA10*, **(B)** *HOXB3*, and **(C)** *HOXC5* gene expression levels on microarray.

**Figure 2:**
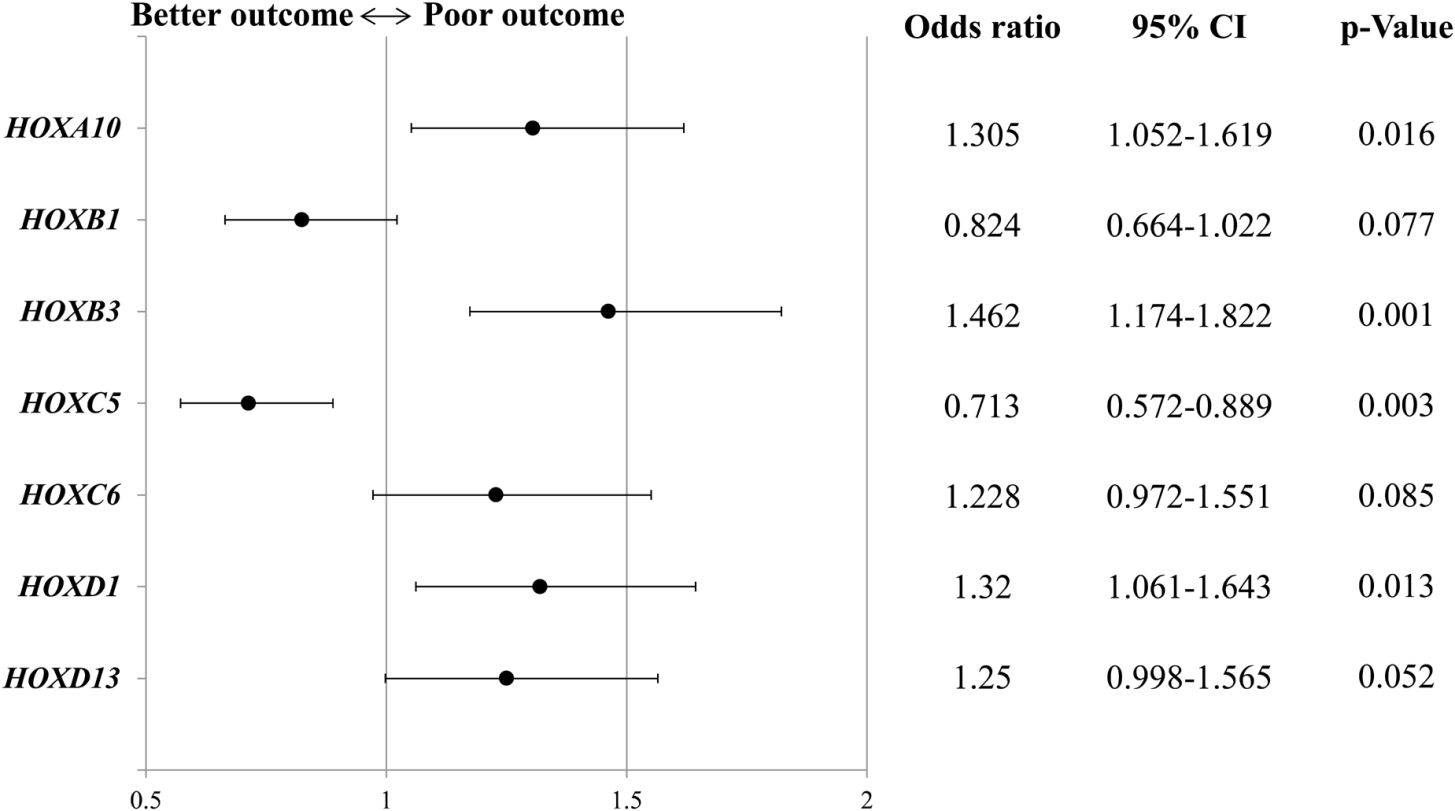
Multivariate analyses of *homeobox (HOX)* gene expression predicting overall survival CI, confidence interval.

Out of the total 630 patients, 584 patients (92.7%) had information on both prognostic value and microarray for the transcriptome analysis, so the prognosis of the 584 patients was analyzed. Kaplan-Meier survival analysis showed that overall survival (OS) was significantly shorter in patients with high rather than low expression levels of *HOXA10* (P = 0.008, median OS: 36 vs. 32 months) and *HOXB3* (P = 0.003, median OS: 34 vs. 32 months), and significantly longer in patients with high rather than low *HOXC5* expression (P = 0.022, median OS: 33 vs. 35 months) (Figure 1). Multivariate Cox regression analysis of *HOX* gene expression also demonstrated that *HOXA10* and *HOXB3* were significant positive predictive factors [odds ratio (OR): 1.305 and 1.462, 95% confidence interval (CI): 1.052–1.619 and 1.174–1.822, respectively], while *HOXC5* (OR: 0.713, 95% CI: 0.572–0.889) was a significant negative predictive factor for overall survival (Figure 2).

## DISCUSSION

In this study, we demonstrated that increased mRNA expression of certain *HOX* genes was independently associated with multiple risk factors, including advanced FIGO stage, suboptimal residual disease, and poor performance status, that were found to influence survival outcomes in ovarian cancer using the TCGA database. Specifically, upregulation of *HOXA10* and *HOXB3* genes and downregulation of *HOXC5* were significantly associated with unfavorable survival outcomes in patients with ovarian cancer.

Accumulating evidence indicates that *HOX* genes may be useful biomarkers to predict and evaluate responses to chemotherapy [17]. Our results were not completely consistent with those of previous *in vitro* research. In particular, the clinical relevance of *HOXB3* and *HOXC5* was not known until now. This might be explained by the fact that the majority of studies reported to date have been retrospective in nature or conducted in small sized patient cohorts. To the best of our knowledge, this is the first study of *HOX* genes performed using clinical data from TCGA database. Also, it is feasible to clinically classify each subtype to determine the prognosis based on the results of this study. Further functional studies are required to identify the specific mechanisms by which *HOX* gene dysregulation influences oncogenesis.

During embryogenesis, as well as in adult tissues, growth, differentiation, and organization of cell populations are tightly coordinated and controlled [18]. In contrast, cancer has been identified as aberrations of these basic processes [19]. The oncogerminative theory of carcinogenesis suggests that malignant transformation is due to the aberrant expression of embryogenesis-related genes [20]. According to this concept, carcinogenesis is a dynamic self-organizing process that is similar to the process of early embryo development. Additionally, the malignant transformation that arises from gene mutations, in combination with epigenetic dysregulation, may reprogram somatic cells into immortal cells that simulate germline cells; this is consistent with cancer stem cells [20].

A substantial body of evidence supports a crucial role for *HOX* genes as developmental genes during embryogenesis, and also supports the critical role of aberrant *HOX* gene expression in the development of various tumors, specifically in ovarian cancer [5–7, 17, 21, 22]. Further research is needed to elucidate all roles of *HOX* genes in ovarian cancer. A major limitation of this study is the short-term follow-up period, and the small number of overall mortalities during the period might weaken the clinical applicability of the current findings. A more comprehensive investigation is promising in the further analysis based on the regularly updated TCGA data in the future. Although this study proved the hypothesis that dysregulated expression of *HOX* genes, based on microarray data, was correlated with altered survival outcomes in EOC, further validation using a different modality, such as large-volume RNA sequencing or immunohistochemical assay, might also be needed to verify the current results.

Analyzing *HOX* gene expression levels in a cytologic or surgical specimen can facilitate the selection of patients with ovarian cancer who are expected to have a poor prognosis. Based on analytical results, we can prescribe more aggressive treatment or more frequent follow-up for cases with an expected high risk. Moreover, there are great unmet needs of patients with refractory ovarian cancer that recurs after treatment with standard therapy, which demands novel therapeutic agents. Drugs regulating *HOX* gene expression could potentially have a therapeutic effect by inducing inhibition of reprogramed oncogerminative cells, as was shown by *in vitro* experiments using a homeobox transcription factor inhibitor [23]. Recently, decitabine, a drug that induced CpG island hypomethylation of *HOXA10* and *HOXA11* genes, showed significant correlation with improved progression-free survival in an open-label, single-center, phase II clinical trial [24].

In conclusion, dysregulated expression of the *HOXA10*, *HOXB3*, and *HOXC5* genes was significantly associated with favorable or unfavorable overall survival in a large ovarian cancer cohort from TCGA. Additionally, many of the *HOX* genes were correlated with clinical variables that were independent predictive factors for overall survival. Evaluation of *HOX* gene expression may be potentially valuable as a marker to predict prognosis in serous ovarian cancer.

## MATERIALS AND METHODS

### Data acquisition

We collected mRNA expression data for 39 *HOX* family genes and corresponding clinicopathological information from the TCGA data portal (https://tcga-data.nci.nih.gov/tcga/tcgaDownload.jsp). The patient personal information is anonymized and deidentified. According to the TCGA publication guidelines (http://cancergenome.nih.gov/publications/publicationguidelines), these mRNA sequencing data have no restrictions on publication, and no additional approval by an ethics committee was required to publish findings utilizing the data. The database compiled from microarray analysis performed using GeneChip Human Genome U133A 2.0 Array (Affymetrix) was analyzed in this study.

### Patient information

We obtained the mRNA expression levels of 39 *HOX* family genes for each patient, along with their clinicopathologic features, including age at initial pathologic diagnosis, International FIGO stage, histologic subtype, lymph node metastasis, and overall survival (Supplementary Table 1).

### Statistical analyses

We used the Fisher exact or χ2 tests for categorical variables, according to sample size. Linear regression was applied to assess associations between clinical variables and each *HOX* gene expression count. Medians of *HOX* gene expression were determined as the cut-off value for prediction of survival. Kaplan-Meier survival analyses and multivariate Cox regression analysis based on the calculated cut-off values were performed. SPSS version 23.0 (IBM Corp., Armonk, NY, USA) was used for data analysis.

## SUPPLEMENTARY MATERIALS TABLE





## Abbreviations

HOX: homeobox
TCGA: The Cancer Genome Atlas
EOC: epithelial ovarian cancer
OR: odds ratio
CI: confidence interval
FIGO: Federation of Gynecology and Obstetrics
N/A: not available
ECOG: The Eastern Cooperative Oncology Group performance status
