# Supplementary material for: Dysregulated expression of homeobox family genes may influence survival outcomes of patients with epithelial ovarian cancer: analysis of data from The Cancer Genome Atlas
Source: Oncotarget. 2017 Aug 1;8(41):70579–85. doi: 10.18632/oncotarget.19771 (PMC5642578; doi:10.18632/oncotarget.19771)
Supplement: Supplementary file 1 [file oncotarget-08-70579-s001.pdf]

Dysregulated expression of *homeobox* family genes may influence survival outcomes of patients with epithelial ovarian cancer: analysis of data from The Cancer Genome Atlas

SUPPLEMENTARY MATERIALS

Supplementary Table 1: Patient clinicopathologic information with mRNA expression levels of HOX family genes

See Supplementary File 1
